# Conformational Restriction of Histamine with a Rigid Bicyclo[3.1.0]hexane Scaffold Provided Selective H_3_ Receptor Ligands

**DOI:** 10.3390/molecules25163562

**Published:** 2020-08-05

**Authors:** Mizuki Watanabe, Takaaki Kobayashi, Yoshihiko Ito, Shizuo Yamada, Satoshi Shuto

**Affiliations:** 1 Faculty of Pharmaceutical Sciences, Hokkaido University, Kita-12, Nishi-6, Kita-ku, Sapporo 060–0812, Japan; kobayashit27@sc.sumitomo-chem.co.jp; 2 Center for Pharma-Food Research (CPFR), Graduate School of Pharmaceutical Sciences, University of Shizuoka, 52-1, Yada, Suruga-ku, Shizuoka 422–8526, Japan; y-ito@u-shizuoka-ken.ac.jp (Y.I.); yamada@u-shizuoka-ken.ac.jp (S.Y.); 3 Center for Research and Education on Drug Discovery, Hokkaido University, Kita-12, Nishi-6, Kita-ku, Sapporo 060–0812, Japan

**Keywords:** histamine, H_3_ receptor, conformational restriction, selective ligands

## Abstract

We designed and synthesized conformationally rigid histamine analogues with a bicyclo[3.1.0]hexane scaffold. All the compounds were selectively bound to the H_3_ receptor subtype over the H_4_ receptor subtype. Notably, compound **7** showed potent binding affinity and over 100-fold selectivity for the H_3_ receptors (*K*_i_ = 5.6 nM for H_3_ and 602 nM for H_4_). These results suggest that the conformationally rigid bicyclo[3.1.0]hexane structure can be a useful scaffold for developing potent ligands selective for the target biomolecules.

## 1. Introduction

**Figure 1:**
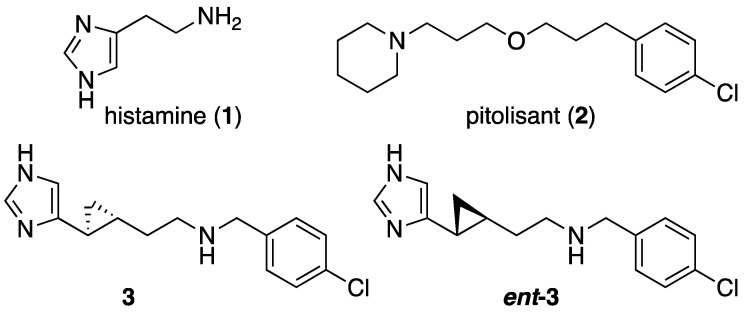
Chemical structures of histamine (**1**), pitolisant (**2**), and cyclopropane-based conformationally restricted histamine analogues as H_3_/H_4_ antagonists (**3** and ***ent*-3**).

Histamine (**1**, Figure 1), a monoamine chemical mediator, acts on four different receptor subtypes, H_1_, H_2_, H_3_, and H_4_ receptors, which are members of the G protein-coupled receptor superfamily. In early the 1980s, the histamine H_3_ receptor (H_3_R) was identified as a presynaptic autoreceptor in rats [1], and the human H_3_R was cloned in 1999 [2]. The H_3_R, which is mainly expressed in the central nervous system (CNS), not only acts as an autoreceptor to regulate the central levels of histamine but also acts as a heteroreceptor to regulate the synthesis and release of other neurotransmitters, such as noradrenaline, acetylcholine, dopamine, and serotonin, and it is involved in various physiological processes, including memory function, cognition, anxiety, pain, food intake, and body temperature regulation. Thus, H_3_R activity inhibition could be a potential treatment for various CNS-related disorders, such as attention-deficit hyperactivity disorder, Alzheimer’s disease, narcolepsy, Parkinson’s disease, schizophrenia, and obesity. Much effort has been devoted to developing potent and selective H_3_R inhibitors (antagonists/inverse agonists) [3,4,5], and pitolisant (**2**, Figure 1) was recently authorized in Europe as a medicine for treating narcolepsy with or without cataplexy [6]. Further research is needed to better understand the functions of the histaminergic system in CNS-related disorders and to advance useful H_3_ antagonists into clinical trials and the market [7].

The development of efficient H_3_-selective antagonists is often complicated by the high affinity of H_3_ antagonists for the H_4_ receptor (H_4_R) because the structures of the two receptor subtypes are presumed to be analogous due to their highly homologous gene sequences (58% identity in the transmembrane regions) [8,9]. However, precise three-dimensional structural information of H_3_R and H_4_R is unavailable, because their X-ray crystal structures have not been reported. To efficiently develop bioactive ligands, even if the structural information of the target proteins is unknown, we developed a three-dimensional structural diversity-oriented strategy based on the characteristic structural properties of chiral cyclopropane, which is the rigid and smallest carbocyclic structure [10]. Introducing cyclopropane into the conformationally flexible acyclic backbone of a parent compound can restrict the conformation of the molecule in either its *trans-* or *cis*-form. That is, four stereoisomer derivatives, including the enantiomers, can be produced from one parent ligand, in which the functional groups essential for the binding to a target protein can be restricted in different spatial arrangements. Therefore, one restricted structure of these isomers may be analogous to the bioactive form of the parent ligand, resulting in the discovery of a lead compound that is superior to the prototype ligand in its target selectivity and/or binding potency [11,12,13,14,15]. Based on this strategy, we previously designed and synthesized a series of cyclopropane-based conformationally restricted histamine analogues having an imidazolylcyclopropane scaffold and identified **3** and its enantiomer ***ent*****-3** as potent H_3_ antagonists (*K*_i_: 3.6 nM and 8.4 nM for the H_3_R, respectively, shown in Figure 1) [16]. These antagonists have high binding affinities for the H_3_R; however, the selectivity between H_3_R and H_4_R is insufficient with selectivity ratios for H_4_/H_3_ of 10.3 (**3**) and 0.9 (***ent*-3**). Thus, in this study, to obtain more highly selective ligands for H_3_R, we adopted bicyclo[3.1.0]hexane as a more rigid scaffold for newly designed conformationally restricted histamine analogues [17]. 

## 2. Results

### 2.1. Design

**Figure 2:**
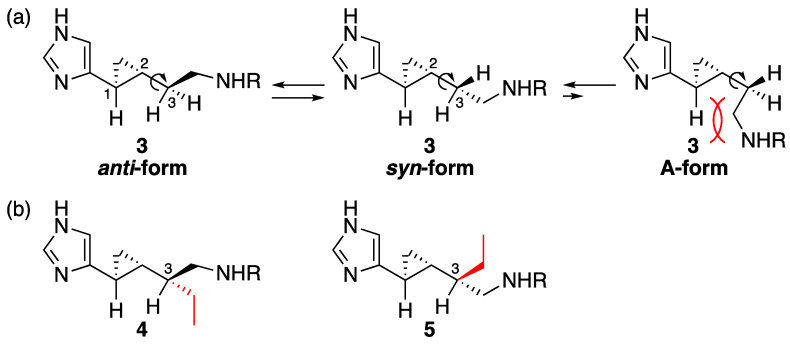
(**a**) The *anti*-, *syn*- and A-forms in **3**; (**b**) The conformationally restricted histamine analogues **4** and **5** with cyclopropylic strain. R indicates a 4-chlorobenzyl group.

We assumed that the reason for the low H_4_/H_3_ selectivity of **3** and ***ent*-3** was the flexibility of the aminoalkyl side chain in the compounds. Although the backbone conformation is restricted to the *trans*-configuration, there are two conformers, *anti*- and *syn*-form, whose aminoalkyl chains orient to the side opposite and same to the cyclopropane ring, respectively, due to the C2–C3 bond rotation (Figure 2a). This bond rotation is limited by the “cyclopropylic strain” that causes a sizable steric repulsion similar to the 1,3-allylic strain between the two adjacent *cis*-configured substituents, which are fixed in an eclipse orientation on the cyclopropane ring. The stable conformation is where the smallest group on the C3 position orients inward to the cyclopropane ring. Conformational analysis using MacroModel 8.6 showed that the *anti*- and *syn*-forms of **3** are the two most stable conformations (Δ*E* = 0.3 kcal/mol), whereas the A-form of **3** is extremely unstable due to the cyclopropylic strain (Δ*E* > 3 kcal/mol, relative potential energy from the global minimum; Figure 2a).

We designed and synthesized the C3-ethyl-substituted derivatives **4** and **5**, as side-chain conformation-restricted analogues of **3** (Figure 2b) [18]. Introducing an ethyl group into the C3 position of **3** prevents the rotation of the side aminoalkyl-chain moiety to effectively restrict the conformation due to the cyclopropylic strain [10]. Accordingly, depending on the configuration at the C3 position, the conformation of the compounds can be restricted to the *anti*-form in **4** and the *syn-*form in **5**. The biological evaluation for H_3_R demonstrated that the *anti*-restricted **4** exhibited high binding affinity for H_3_R (*K*_i_ = 6.7 nM) comparable to **3**; on the other hand, the binding affinity of *syn*-restricted **5** for H_3_R (*K*_i_ = 63.0 nM) was more than 15-fold lower than that of **3**. These results showed that the conformational restriction of the side chain to the *anti*- or *syn*-form affects the binding affinity to the target protein. Unfortunately, however, further biological assay showed that **4** and **5** not only bound to H_3_R but also to H_4_R and were non-selective for the two receptor subtypes (selectivity ratios for H_4_/H_3_ of 1.2–1.3, Table 1).

**Figure 3:**
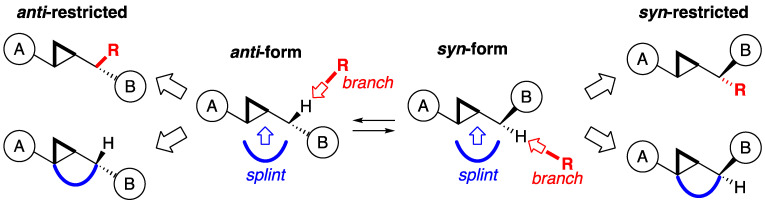
The two strategies to conformationally restrict the cyclopropane-based analogues to the *anti*- or *syn*-form by the introduction of a “branch” substituent inducing the cyclopropylic strain or a “splint” moiety forming the bicyclo structure.

**Figure 4:**
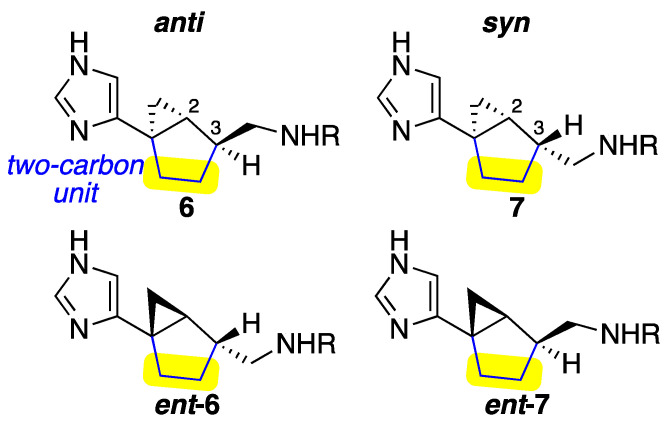
The conformationally restricted histamine analogues **6** and **7** with a bicyclo[3.1.0]hexane backbone, and their enantiomers ***ent*-6** and ***ent*-7**. R indicates a 4-chlorobenzyl group.

Taking these results into account, we planned to obtain highly selective ligands for H_3_R by adopting an alternative strategy that utilizes a rigid fused-ring structure as a scaffold (Figure 3). Cyclopropane-based conformationally restricted ligands have two side-chain orientations, *anti* and *syn*, as described above. The introduction of a "branch" substituent on the cyclopropane adjacent carbon in the side-chain allows the orientation to be restricted to *anti* or *syn* due to the cyclopropylic strain, depending on the configuration at the cyclopropane adjacent carbon. On the other hand, instead of utilizing the cyclopropylic strain, the side-chain orientation can be restricted to *anti* or *syn* by introducing a “splint” structure between the cyclopropane and the side chain to form a rigid bicyclo structure. Both of these different conformational restriction strategies can generate the *anti*- and *syn*-restricted cyclopropane derivatives. However, even if they are the same *anti*- or *syn*-restricted analogues, the difference in the branch and splint units introduced to regulate the conformation may cause different biological profiles. Therefore, in this study, we newly designed conformationally restricted histamine analogues **6**, **7**, and their enantiomers ***ent*-6**, ***ent*-7** with a bicyclo[3.1.0]hexane scaffold that can restrict the spatial arrangement of the imidazole and the aminoalkyl chain (Figure 4). The aminoalkyl chains of **6** and **7** were restricted to *anti* or *syn*, respectively, because the splint “two-carbon unit” forming the bicyclo backbone can lock the C2–C3 bond rotation. We previously used the bicyclo[3.1.0]hexane structure as a backbone for conformationally restricted analogues of γ-aminobutyric acid (GABA) and reported the first potent and selective inhibitor of BGT-1, which is a GABA transporter subtype [19]. Thus, we expected that the conformationally restricted histamine analogues with the bicyclo[3.1.0]hexane scaffold would show selectivity for H_3_R, in contrast to the previous cyclopropylic strain-based conformationally restricted analogues.

### 2.2. Chemistry

**Scheme 1:**
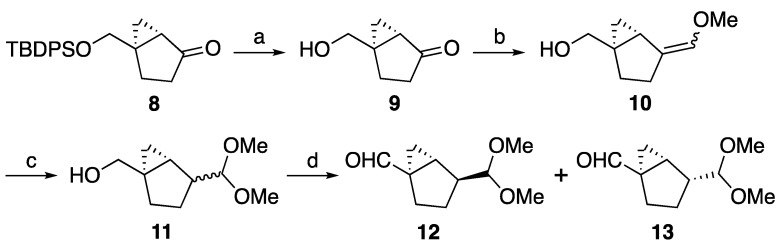
Reagents and conditions. (**a**) 3HF•Et_3_N, THF, 90%; (**b**) MeOCH_2_PPh_3_Cl, *t*BuOK, THF, 0 °C, 90%; (**c**) TsOH•H_2_O, MeOH, reflux, 85%; (**d**) SO_3_•pyridine, Et_3_N, DMSO, 29% (**12**) and 52% (**13**).

The diastereomerically pure bicyclo[3.1.0]hexane **12** and **13** with all of the asymmetric centers in target compounds **6** and **7**, respectively, were synthesized from ketone **8** [19], which was prepared from (*S*)-epichlorohydrin, as shown in Scheme 1. After removing the *O*-silyl protecting group of **8**, one-carbon addition to **9** was conducted by Wittig reaction to give olefin **10** as an *E*/*Z* mixture. The treatment of **10** with *p*-toluenesulfonic acid in MeOH under reflux conditions afforded dimethyl acetal **11** as a diastereomeric mixture. Oxidation of the hydroxyl group of **11** and final separation by silica gel column chromatography gave the aldehyde isomers **12** and **13**, respectively.

**Scheme 2:**
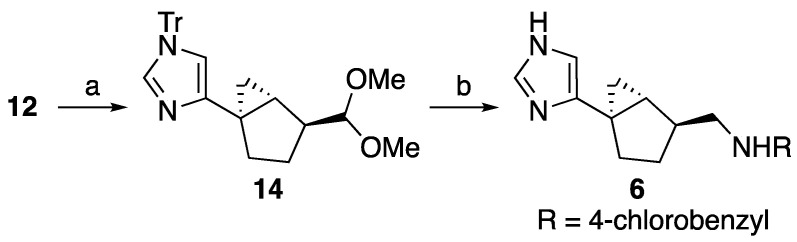
Reagents and conditions. (**a**) (1) TsCH_2_NC, NaOEt, EtOH, 0 °C; (2) sat. NH_3_/EtOH, 125 °C, sealed tube; (3) TrCl, Et_3_N, CH_2_Cl_2_, 69% in 3 steps; (**b**) (1) HCO_2_H, hexane (2) 4-chlorobenzyl amine, NaBH(OAc)_3_, MS4Å, CH_2_Cl_2_; (3) Boc_2_O, Et_3_N, DMAP, MeOH; (4) aq. HCl, EtOH, reflux, 49% in 4 steps.

The conformationally restricted histamine analogue **6** was synthesized from **12** (Scheme 2). The imidazole ring was constructed by treating **12** with tosylmethyl isocyanide, followed by saturated ethanolic ammonia [20]. The resulting unpurified imidazole product was further treated with trityl chloride to afford *N*-tritylimidazolylcyclopropane **14** in 69% overall yield. Removal of the dimethyl acetal group under acidic conditions followed by reductive amination with 4-chlorobenzyl amine gave the desired secondary amine, which was protected by a Boc group for easy purification. Finally, acidic treatment of the protected amine simultaneously removed the *N*-Boc and *N*-Tr groups to provide the desired compound **6**. The diastereomer **7** was similarly prepared from **13** (Scheme S1). Configurations of the C3 position in **6** and **7** were determined based on the nuclear Overhauser effect (NOE) difference spectra (Figure S1). In addition, enantiomers ***ent*-6** and ***ent*-7** were synthesized by the same procedure from ***ent*-8**, which was prepared from (*R*)-epichlorohydrin (Scheme S2).

### 2.3. Biological Evaluation

The binding affinities of **6**, **7**, ***ent*-6**, and ***ent*-7** for the human H_3_R subtype with [^3^H]*N*^α^-methylhistamine and the human H_4_R subtype with [^3^H]histamine were evaluated using cell membranes expressing human H_3_R or H_4_R according to the previously reported method, and the results are summarized in Table 1. These conformationally locked compounds, **6**, **7**, ***ent*-6**, and ***ent*-7** had potent binding affinities for H_3_R, although their *K*_i_ values were slightly larger than those of their parent compounds **3** or ***ent*-3** [*K*_i_: **6** (31.6 nM), **7** (5.6 nM) versus **3** (3.6 nM) and ***ent*-6** (21.9 nM), ***ent*-7** (14.8 nM) versus ***ent*-3** (8.4 nM)]. On the other hand, their *K*_i_ values for H_4_R were much larger than those of the parents [*K*_i_: **6** (501 nM), **7** (602 nM) versus **3** (37.2 nM) and ***ent*-6** (416 nM), ***ent*-7** (69.2 nM) versus ***ent*-3** (7.6 nM)]. Thus, compared to the parents, the bicyclo compounds exhibited clearly improved selectivity for H_3_R. Notably, **7** showed highly potent binding affinity and more than 100-fold selectivity for H_3_R over the H_4_R. 

## 3. Discussion

**Figure 5:**
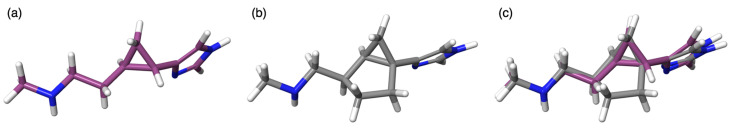
The most stable conformation of the *syn*-form in **3** (**a**) and **7** (**b**) by conformational search using MacroModel 10.9. (force field: MMFFs, solvent: H_2_O); (**c**) Superimposition of the stable conformation of **3** and **7**. The chlorobenzyl moiety was replaced with a methyl group to simplify the structures.

Comparison of the most stable conformations of **7** and the *syn*-form in **3** that were obtained by the MacroModel calculations revealed that the imidazole and amino moieties, which are essential for binding to the receptors, are superimposable; however, the splint “two-carbon unit” introduced for forming the bicyclo backbone of **7** is rather bulky and protrudes from the backbone of **3** (Figure 5). Therefore, the higher H_3_R selectivity of the bicyclo compounds than that of the parents (Table 1) suggest that the splint unit in **6** and **7** caused a steric repulsion in the binding mode for H_4_R, resulting in their low affinity for H_4_R. In other words, there seems to be a difference in the three-dimensional structures between H_3_R and H_4_R around the imidazole binding area in their orthosteric sites.

**Figure 6:**
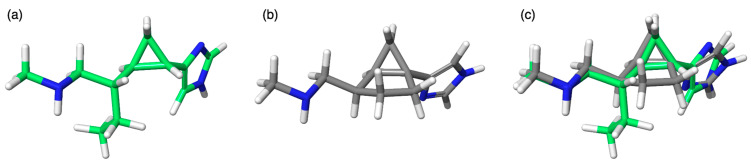
The most stable conformation of **5** (**a**) and **7** (**b**) by conformational search using MacroModel 10.9. (force field: MMFFs, solvent: H_2_O); (**c**) Superimposition of the stable conformation of **5** and **7**. The chlorobenzyl moiety was replaced with a methyl group to simplify the structures.

The *syn*-restricted **7** with the bicyclo scaffold displayed high selectivity for H_3_R, although the similarly *syn*-restricted **5** with a branched ethyl group for the cyclopropylic strain was non-selective for H_3_R and H_4_R (Table 1). Compared with their three-dimensional structures, the most stable conformations of **5** and **7** calculated by MacroModel are superimposable at the essential imidazole and amino functions (Figure 6). However, the “branch” and “splint” substituents, i.e., the ethyl group introduced to restrict the conformation by the cyclopropylic strain in **5** and the “two-carbon unit” moiety to form the bicyclo scaffold in **7**, are positioned differently in space. In addition, in the most stable conformations of *anti*-restricted **4** and **6**, the imidazole and amino functions are superimposable, but the branch and splint moieties are differently positioned (Figure S3). These results indicate that even when restricted to the same *anti*- or *syn*-form, the “branch” or “splint” moieties that were introduced to restrict the conformation may interact with the target receptor or undergo steric repulsion due to the difference in the spatial arrangement of the moieties, potentially causing the different subtype selectivity. For example, among these four compounds, **4**–**7**, only **4** had a high binding affinity for H_4_R (*K*_i_ = 9.0 nM, Table 1).

The rigid bicyclo[3.1.0]hexane scaffold was effectively used for the conformational restriction of cyclopropane side chains to improve the H_3_R/H_4_R-selectivity, similar to the previous results in the development of a selective inhibitor for the GABA transporter BGT-1 subtype [19]. On the other hand, the cyclopropylic strain-based conformational restriction strategy has successfully provided a variety of compounds of biological interest, such as NMDA receptor antagonists [21], proteasome inhibitors [22], melanocortin receptor antagonists [23], and membrane-permeable cyclic peptides [24]. Thus, the two conformational restriction methods using cyclopropane as the key structure effectively complement each other in medicinal chemistry studies for developing potent and selective ligands for various target proteins. 

## 4. Materials and Methods

### 4.1. Synthesis

All ^1^H-NMR and ^13^C-NMR spectra were recorded on a JEOL JNM-AL-400, JEOL JMM-ECX-400P, or JEOL JMM-ECA-500 spectrometer. ^1^H-NMR chemical shifts are reported as d values in ppm relative to tetramethylsilane (0.00 ppm) when CDCl_3_ was used as the solvent, or a solvent residual peak (CD_2_H-OD: 3.31 ppm) when CD_3_OD was used as the solvent. Coupling constants (*J*) are reported in Hz, and multiplicity is indicated as follows: s (singlet), d (doublet), dd (double doublet), t (triplet), q (quartet), brs (broad singlet). ^13^C-NMR chemical shifts are reported as d values in ppm relative to deuterated solvent (CDCl_3_: 77.0 ppm or CD_3_OD: 49.0 ppm). All mass spectra were obtained on a JEOL JMS-T100LC spectrometer. Elemental analysis was performed with a Yanaco CHN Corder MT-6 or J-Science MICRO CODER JM10 analyzer. Optical rotations were measured with a JASCO P-1030 digital polarimeter. All non-aqueous reactions were carried out under argon atmosphere in anhydrous grade solvents. Silica gel column chromatography was performed using Merck Silica Gel 60, Kanto Silica Gel 60N, or Chromatorex^®^ NH-DM1020 (Fuji Silysia Cheical Ltd.). TLC was performed using glass-backed silica gel 60F254.

*(1S,5S)-1-Hydroxymethyl-bicyclo[3.1.0]hexan-4-one* (**9**). The mixture of **8** (983 mg, 2.70 mmol) and 3HF•Et_3_N (2.2 mL, 6.99 mmol) in THF (25 mL) was stirred at room temperature (rt) for 3 days. The solvent was evaporated, and the residue was purified by flash silica gel column chromatography (hexane/AcOEt, 1/1–0/1) to give **9** (307 mg, 2.43 mmol, 90%) as a colorless liquid. [α]_D_^22^ = +4.1° (*c* 0.90, CHCl_3_); ^1^H-NMR (400 MHz, CDCl_3_) δ 3.82 (1 H, d, *J* = 11.6 Hz, –CHaHbOH), 3.66 (1 H, d, *J* = 11.6 Hz, –CHaHbOH), 2.22–2.09 (4 H, m, H-2, H-3), 1.78 (1 H, dd, *J* = 9.1, 3.2 Hz, H-5), 1.33 (1 H, dd, *J* = 9.1, 5.0 Hz, H-6a), 1.19 (1 H, dd, *J* = 5.0, 3.2 Hz, H-6b); ^13^C NMR (100 MHz, CDCl_3_) δ 214.98, 64.67, 35.92, 33.00, 31.99, 23.83, 17.55; HRMS (ESI) calcd for C_7_H_10_NaO_2_ 149.0573, found 149.0573 [(M + Na)^+^].

*(1S,5S)-1-Hydroxymethyl-4-methoxymethylenebicyclo[3.1.0]hexane* (**10**, *E*/*Z*-mixture). To a suspension of methoxymethyltriphenylphosphonium chloride (2.96 g, 8.52 mmol) in THF (20 mL) was added *t*BuOK (ca. 1.0 M solution in THF, 8.0 mL, 8.0 mmol) at 0 °C, and the mixture was stirred at the same temperature for 1 h. To the reaction mixture was added a solution of **9** (307 mg, 2.43 mmol) in THF (4 mL) at 0 °C, and the resulting mixture was stirred at the same temperature for 4 h. After the addition of sat. aq. NH_4_Cl, the reaction mixture was concentrated in reduced pressure, and the residue was partitioned between AcOEt and sat. aq. NH_4_Cl. The organic layer was washed with brine, dried (Na_2_SO_4_), and evaporated. The residue was purified by silica gel column chromatography (hexane/AcOEt, 50/1) to give **10** (338 mg, 2.19 mmol, 90%, *E*/*Z*-mixture, 1/0.8) as a pale yellow oil. [α]_D_^21^ = +49.6° (*c* 1.00, CHCl_3_); HRMS (ESI) calcd for C_9_H_14_NaO_2_ 177.0886, found 177.0887 [(M + Na)^+^]; Major isomer, ^1^H-NMR (400 MHz, CDCl_3_) δ 5.85 (1 H, s, MeOCH=C–), 3.62 (2 H, s, –CH_2_OH), 3.55 (3 H, s, CH_3_O–), 3.02 (1 H, brs, OH), 2.12 (1 H, m, H-3a), 1.97 (1 H, dd, *J* = 8.1, 3.6 Hz, H-5), 1.93–1.78 (3 H, m, H-2, H-3b), 0.81 (1 H, dd, *J* = 8.1, 4.5 Hz, H-6a), 0.74 (1 H, m, H-6b); ^13^C NMR (100 MHz, CDCl_3_) δ 137.57, 121.41, 67.07, 59.14, 33.11, 28.16, 24.60, 22.69, 14.27; Minor isomer, ^1^H-NMR (400 MHz, CDCl_3_) δ 5.92 (1 H, s, MeOCH=C-), 3.62 (2 H, s, –CH_2_OH), 3.53 (3 H, s, CH_3_O–), 3.02 (1 H, brs, OH), 2.55 (1 H, m, H-3a), 1.93–1.77 (3 H, m, H-2, H-3b), 1.61 (1 H, dd, *J* = 8.1, 3.6 Hz, H-5), 0.73 (1 H, m, H-6a), 0.61 (1 H, m, H-6b). 

*(1S,5R)-4-Dimethoxymethyl-1-hydroxymethylbicyclo[3.1.0]hexane* (**11**, diastereomixture). The mixture of **10** (20 mg, 0.13 mmol) and *p*-toluenesulfonic acid monohydrate (5.0 mg, 0.026 mmol) in MeOH (2 mL) was stirred under reflux conditions for 3 h. After the addition of sat. aq. NaHCO_3_ at 0 °C, the resulting mixture was concentrated in reduced pressure. The residue was partitioned between AcOEt and sat. aq. NaHCO_3_. The organic layer was washed with brine, dried (Na_2_SO_4_), and evaporated. The residue was purified by flash silica gel column chromatography (hexane/AcOEt, 4/1–0/1) to give **11** (20 mg, 0.11 mmol, 85%, diastereomixture, 1/0.8) as a pale yellow liquid. [α]_D_^21^ = +54.1° (*c* 1.18, CHCl_3_); HRMS (ESI) calcd for C_10_H_18_NaO_3_ 209.1148, found 209.1149 [(M + Na)^+^]; Major diastereomer, ^1^H-NMR (400 MHz, CDCl_3_) δ 4.10 (1 H, d, *J* = 9.0 Hz, –CHCH(OMe)_2_), 3.74 (1 H, d, *J* = 11.4 Hz, –CHaHbOH), 3.43 (1 H, d, *J* = 11.4 Hz, –CHaHbOH), 3.39 (3 H, s, –OCH_3_), 3.37 (3 H, s, –OCH_3_), 2.24 (1 H, m, H-4), 1.93 (1 H, m, H-3a), 1.82 (1 H, m, H-2a), 1.77 (1 H, m, H-2b), 1.36 (1 H, m, H-3b), 1.18 (1 H, m, H-5), 0.58 (1 H, m, H-6a), 0.45 (1 H, m, H-6b); ^13^C NMR (100 MHz, CDCl_3_) δ 107.66, 67.42, 52.99, 52.70, 42.27, 30.72, 28.70, 23.70, 22.74, 8.77; Minor diastereomer, ^1^H-NMR (400 MHz, CDCl_3_) δ 4.12 (1 H, d, *J* = 6.7 Hz, –CHCH(OMe)_2_), 3.64–3.57 (2 H, s, –CH_2_OH), 3.39 (3 H, s, –OCH_3_), 3.31 (3 H, s, CH_3_O–), 2.50 (1 H, m, H-4), 1.82 (1 H, m, H-3a), 1.71–1.62 (2 H, m, H-2a), 1.18 (1 H, m, H-5), 1.02 (1 H, m, H-3b), 0.58 (1H, m, H-6a), 0.41 (1 H, m, H-6b); ^13^C NMR (100 MHz, CDCl_3_) δ 107.15, 67.19, 54.19, 53.15, 42.71, 31.13, 27.19, 23.49, 22.54, 11.85.

*(1S,4S,5R)-4-Dimethoxymethyl-1-formylbicyclo[3.1.0]hexane* (**12**, *anti*) and *(1S,4R,5R)-4-Dimethoxymethyl-1-formylbicyclo[3.1.0]hexane* (**13**, *syn*). To a solution of **11** (152 mg, 0.816 mmol) in DMSO (9.0 mL) were added Et_3_N (0.349 mL, 2.46 mmol) and pyridine–SO_3_ complex (262 mg, 1.64 mmol), and the mixture was stirred at rt for 3 h. After the addition of sat. aq. NH_4_Cl, the mixture was partitioned between AcOEt and sat. aq. NH_4_Cl. The organic layer was washed with brine, dried (Na_2_SO_4_), and evaporated. The residue was purified by flash silica gel column chromatography (hexane/AcOEt, 8/1) to give **12** (43 mg, 0.23 mmol, 29%, *anti,* less polar) as a colorless oil and **13** (78 mg, 0.43 mmol, 52%, *syn*, more polar) as a colorless oil. **12**: [α]_D_^21^ = –2.6° (*c* 0.99, CHCl_3_); ^1^H-NMR (400 MHz, CDCl_3_) δ 8.90 (1 H, s, CHO), 3.98 (1 H, d, *J* = 8.2 Hz, –CHCH(OMe)_2_), 3.28 (3 H, s, –OCH_3_), 3.26 (3 H, s, –OCH_3_), 2.26 (1 H, dd, *J* = 8.2, 7.7 Hz, H-4), 2.16 (1 H, m, H-3a), 1.96 (1 H, dd, *J* = 9.1, 5.4 Hz, H-5), 1.73 (1 H, dd, *J* = 14.0, 8.6 Hz, H-2a), 1.64 (1 H, dd, *J* = 13.1, 8.6 Hz, H-5b), 1.40–1.29 (2 H, m, H-3b, H-6a), 1.02 (1 H, dd, *J* = 5.4, 5.4 Hz, H-6b); ^13^C NMR (100 MHz, CDCl_3_) δ 200.19, 105.39, 53.41, 53.34, 42.03, 41.73, 29.44, 23.06, 22.81, 15.68; HRMS (ESI) calcd for C_10_H_16_NaO_3_ 207.0992, found 207.0992 [(M + Na)^+^]; **13**: [α]_D_^21^ = +71.5° (*c* 0.99, CHCl_3_); ^1^H-NMR (400 MHz, CDCl_3_) δ 8.87 (1 H, s, CHO), 4.10 (1 H, d, *J* = 7.7 Hz, –CHCH(OMe)_2_), 3.32 (3 H, s, –OCH_3_), 3.26 (3 H, s, –OCH_3_), 2.48 (1 H, m, H-4), 2.23 (1 H, m, H-3a), 1.90 (1 H, m, H-5), 1.76–1.66 (2 H, m, H-2), 1.26 (1 H, dd, *J* = 8.6, 5.9 Hz, H-6a), 1.16 (1 H, dd, *J* = 5.9, 5.4 Hz, H-6a), 1.05 (1 H, m, H-3b); ^13^C NMR (100 MHz, CDCl_3_) δ 200.12, 106.86, 53.32, 52.81, 41.57, 41.26, 28.71, 23.97, 22.99, 13.24; HRMS (ESI) calcd for C_10_H_16_NaO_3_ 207.0992, found 207.0993 [(M + Na)^+^].

*(1S,4S,5R)-4-Dimethoxymethyl-1-(1-triphenylmethyl-1H-imidazol-4-yl)bicyclo[3.1.0]hexane* (**14**). To a suspension of **12** (43 mg, 0.23 mmol) and tosylmethylisocyanide (50 mg, 0.26 mmol) in EtOH (2.3 mL) was added NaOEt (ca. 20% solution in EtOH, 23 µL, 0.053 mmol) at 0 °C, and the mixture was stirred at the same temperature for 3 h. To the reaction mixture was added a saturated NH_3_ solution in EtOH (6 mL) at 0 °C, and the resulting mixture was heated at 125 °C for 20 h in a sealed tube. After being cooled to rt, the reaction mixture was concentrated in reduced pressure. The residue was dissolved in CH_2_Cl_2_ (3.2 mL), and to the solution were added Et_3_N (96 µL, 0.69 mmol) and TrCl (128 mg, 0.46 mmol). The resulting mixture was stirred at rt for 5 h. After the addition of MeOH, the mixture was partitioned between AcOEt and sat. aq. NaHCO_3_. The organic layer was washed with brine, dried (Na_2_SO_4_), and evaporated. The residue was purified by flash silica gel column chromatography (hexane/AcOEt, 10/1–3/1–1/1) to give **14** (75 mg, 0.12 mmol, 69% in 3 steps) as a pale yellow oil. ^1^H-NMR (400 MHz, CDCl_3_) δ 7.32–7.31 (10 H, m, aromatic), 7.15–7.13 (6 H, m, aromatic), 6.54 (1 H, s, imidazole-5), 4.15 (1 H, d, *J* = 8.1 Hz, –CHCH(OMe)_2_), 3.32 (3 H, s, –OCH_3_), 3.30 (3 H, s, –OCH_3_), 2.30 (1 H, dd, *J* = 8.1, 7.6 Hz, H-4), 2.02 (1 H, m, H-3a), 1.85 (1 H, dd, *J* = 12.1, 8.3 Hz, H-2a), 1.72 (1 H, dd, *J* = 13.9, 8.3 Hz, H-2b), 1.63 (1 H, m, H-5), 1.39 (1 H, m, H-3b), 1.15 (1 H, dd, *J* = 8.1, 4.5 Hz, H-6a), 0.72 (1 H, dd, *J* = 4.5, 4.5 Hz, H-6b); ^13^C NMR (100 MHz, CDCl_3_) δ 144.6, 142.4, 138.2, 130.0, 129.7, 127.9, 116.3, 106.1, 75.0, 53.5, 52.8, 43.7, 29.0, 27.9, 27.0, 22.9, 14.6; HRMS (ESI) calcd for C_31_H_32_N_2_NaO_2_ 487.2356, found 487.2368 [(M + Na)^+^].

*(1S,4S,5R)-4-[N-(4-Chlorobenzyl)aminomethyl]-1-(1H-imidazol-4-yl)bicyclo[3.1.0]hexane dihydrochloride* (**6**•**2HCl**). To a suspension of **14** (49 mg, 0.105 mmol) in hexane (0.20 mL) was added formic acid (0.80 mL), and the mixture was stirred at rt for 3 h. After the addition of sat. aq. NaHCO_3_ at 0 °C, the mixture was partitioned between AcOEt and sat. aq. NaHCO_3_. The organic layer was washed with brine, dried (Na_2_SO_4_), and evaporated. The residue was dissolved in CH_2_Cl_2_ (3.5 mL), and to the solution was added 4-chlorobenzylamine (0.038 mL, 0.32 mmol), molecular sieves 4Å (powder, 40 mg), and sodium NaBH(OAc)_3_ (27 mg, 0.13 mmol), and the mixture was stirred at rt for 15 h. After the addition of sat. aq. NaHCO_3_, the mixture was partitioned between AcOEt and sat. aq. NaHCO_3_. The organic layer was washed with brine, dried (Na_2_SO_4_), and evaporated. The residue was passed through silica gel column chromatography (CHCl_3_/MeOH, 1/0–50/1–9/1) to give a corresponding secondary amine. To a solution of the amine in MeOH (1.1 mL) were added Et_3_N (0.088 mL, 0.63 mmol), *N*,*N*-dimethyl-4-aminopyridine (DMAP, 3 mg, 0.025 mmol), and Boc_2_O (0.113 mL, 0.54 mmol), and the mixture was stirred at rt for 5 h. The reaction mixture was concentrated in reduced pressure, and the residue was purified by flash silica gel column chromatography (hexane/AcOEt, 15/1–2/1) to give a corresponding *N*-Boc-amine (33 mg, 0.051 mmol) as a pale yellow oil. To a solution of *N*-Boc-amine (33 mg) in EtOH (1.1 mL) was added aq. HCl (12 M, 0.4 mL), and the mixture was stirred under reflux conditions for 3 h. After being cooled to rt, the reaction mixture was concentrated in reduced pressure. The residue was partitioned between aq. HCl (2 M) and CH_2_Cl_2_. The aqueous layer was neutralized by aq. NaOH (2 M) and partitioned between aq. NaOH (2 M) and CH_2_Cl_2_. The organic layer was washed with brine, dried (Na_2_SO_4_), and evaporated. The residue was purified by NH silica gel column chromatography (CHCl_3_/MeOH, 1/0–50/1) to give **6** as a free amine. **6** was dissolved in a solution of HCl in MeOH (2 M), and the mixture was concentrated in reduced pressure. The residue was triturated with Et_2_O to give **6•2HCl** (19 mg, 0.051 mmol, 49% in 4 steps) as a hygroscopic white solid. [α]_D_^29^ = +16.0° (*c* 0.66, CH_3_OH); ^1^H-NMR (400 MHz, CD_3_OD) δ 8.81 (1 H, s, imidazole-2), 7.62–7.61 (2 H, d, *J* = 8.0 Hz, aromatic), 7.47–7.43 (3 H, m, aromatic and imidazole-5), 4.28 (2 H, s, benzyl), 3.19 (1 H, m, –CHCHaHbN–), 3.11 (1 H, m, –CHCHaHbN–), 2.54 (1 H, m, H-4), 2.23–2.08 (2 H, m, H-2a, and H-3a), 1.86 (1 H, m, H-5), 1.78 (1 H, m, H-2b), 1.66 (1 H, m, H-3b), 1.13 (2 H, m, H-6); ^13^C NMR (100 MHz, CD_3_OD) δ 138.04, 136.66, 134.60. 133.13, 131.22, 130.25, 116.54, 52.43, 52.11, 39.05, 30.29, 29.85, 25.57, 24.42, 15.19; HRMS (ESI) calcd for C_17_H_21_N_3_Cl 302.1419, found 302.1420 [(M + H)^+^]; Anal. Calcd for C_17_H_20_N_3_Cl•2.1HCl: C, 53.96; H, 5.89; N, 11.11. Found: C, 53.90; H, 5.85; N, 11.07.

### 4.2. Biological Assay

The protocol of the biological assay was according to the previous report [16]. The membrane preparations of Chinese hamster ovary (CHO) cells, which expressed recombinant human histamine H_3_ or H_4_ receptors, were purchased from Euroscreen (Brussels, Belgium). The binding assay of the H_3_ and H_4_ receptors was performed using [^3^H]*N*^α^-methylhistamine (Perkin-Elmer, Boston, MA) and [^3^H]histamine (Perkin-Elmer), respectively. Briefly, the membrane preparations (7.5–15 µg protein) were incubated with different concentrations of [^3^H]*N*^α^-methylhistamine (0.1–3 nM) and of [^3^H]histamine(1–30 nM) for 30 min at 25 °C in 50 mM Tris/5 mM MgCl_2_ buffer (pH 7.4). The reaction was terminated by rapid filtration (Cell Harvester, Brandel Co., Gaithersburg, MD) through Whatman GF/B glass fiber filters presoaked for 2 h in 0.5% polyethyleneimine, and the filters were rinsed three times with an ice-cold buffer (2 mL). Membrane-bound radioactivity was extracted from filters overnight in scintillation fluid (toluene, 2 L; Triton X-100, 1 L; 2,5-diphenyloxazole, 15 g; 1,4-bis[2-(5-phenyloxazolyl)]benzene, 0.3 g) and determined in a liquid scintillation counter. The specific binding of each radioligand was determined experimentally from the difference between counts in the presence of 10 µM thioperamide. The apparent dissociation constants (*K*_d_) for each radioligand was determined by nonlinear regression analysis of the curve generated by plotting specific binding concentration versus concentration of the radioligand with GraphPad Prism (GraphPad Software, San Diego, CA) using a one-site binding curve equation. The ability of each compound to inhibit the specific binding of [^3^H]*N*^α^-methylhistamine (1.5 nM) and [^3^H]histamine (25 nM) was estimated by IC_50_ values, which are the molar concentrations of unlabeled drugs necessary for displacing 50% of specific binding (estimated by log probit analysis). The inhibition constant, *K*_i_, was calculated from the equation, *K*_i_ = IC_50_/(1 + *L*/*K*_d_), where *L* equals the concentration of each radioligand. The data were presented as mean ± SE (n = 3–5).

## 5. Conclusions

The imidazolyl bicyclo[3.1.0]hexane derivatives as conformationally restricted histamine analogues designed and synthesized in this study showed binding affinity selective for H_3_R over H_4_R, while the parent imidazolylcyclopropane derivatives showed no selectivity. Notably, compound **7** exhibited over 100-fold H_3_R selectivity. The findings of the present study suggest that the imidazolyl bicyclo[3.1.0]hexane structure is a more useful scaffold than the imidazolylcyclopropane structure for the development of selective H_3_ receptor ligands. Using both conformationally restricted structures, bicyclo[3.1.0]hexane and cyclopropane with cyclopropylic strain, could allow us to produce more bioactive compounds for structurally unknown proteins, e.g., histamine H_3_ and H_4_ receptors, effectively.

## Figures and Tables

**Table 1:** Binding affinities of the compounds for the human H_3_ and H_4_ receptor subtypes.^a^

Compound	Form	H_3_, *K*i (nM)	H_4_, *K*i (nM)	Selectivity Ratio (H_4_/H_3_)
**6**	*anti*	31.6 ± 8.2	501 ± 201	16
**7**	*syn*	5.6 ± 1.6	602 ± 343	108
***ent*-6**	*anti*	21.9 ± 6.0	416 ± 155	19
***ent*-7**	*syn*	14.8 ± 3.8	69.2 ± 21	4.7
**3 ^b^**	*–*	3.6 ± 0.4	37.2 ± 2.7	10
***ent*-3 ^b^**	*–*	8.4 ± 1.5	7.6 ± 0.4	0.9
**4**	*anti*	6.7 ± 0.4 ^c^	9.0 ± 1.0	1.3
**5**	*syn*	63.0 ± 6.1 ^c^	76.5 ± 14	1.2
Thioperamide ^b^	*–*	51 ± 3.8	124 ± 14	2.4

^a^ Data are expressed as means ± SEM (n = 3–5). ^b,c^ Data were taken from [16] (b) and [18] (c).

## Supplementary Materials

The followings are available online: Figures S1–S3; Schemes S1 and S2; Synthetic procedures and characterization of compounds **7**, ***ent*-6**, and ***ent*-7**; ^1^H-NMR spectra of compounds **6**, **7**, ***ent*-6**, and ***ent*-7**.
