# Supplementary material for: Conformational Restriction of Histamine with a Rigid Bicyclo[3.1.0]hexane Scaffold Provided Selective H3 Receptor Ligands
Source: Molecules. 2020 Aug 5;25(16):3562. doi: 10.3390/molecules25163562 (PMC7463632; doi:10.3390/molecules25163562)
Supplement: Supplementary file 1 [file molecules-25-03562-s001.pdf]

Supplementary Materials

Conformational restriction of histamine with a rigid bicyclo[3.1.0]hexane scaffold provided selective H_3 receptor ligands

Mizuki Watanabe [1,*], Takaaki Kobayashi [1], Yoshihiko Ito [2], Shizuo Yamada [2] and Satoshi Shuto [1,3,*]

[1] Faculty of Pharmaceutical Sciences and [3]Center for Research and Education on Drug Discovery, Hokkaido University, Kita-12, Nishi-6, Kita-ku, Sapporo 060–0812, Japan

[2] Center for Pharma-Food Research (CPFR), Graduate School of Pharmaceutical Sciences, University of Shizuoka, 52-1, Yada, Suruga-ku, Shizuoka 422–8526, Japan

*Corresponding authors.

E-mails: mwatanab@pharm.hokudai.ac.jp (M.W.) and shu@pharm.hokudai.ac.jp (S.S.)

Contents

The observed NOEs in compounds **6** and **7**……………………………………………………………S2

Chemical structure of thioperamide………………………………………………………………S2

The most stable conformation of **4** and **6** (Figure S3)….…..…………………………………………….....S2

Synthetic procedures and characterization of the compounds………………………………………...…S3–S5

Reference……………………………………………………………………………………….....S5

^{1}H NMR spectra of compounds **6**, ***ent*-6**, **7**, and ***ent*-7**……………………………......................……......S6–S9

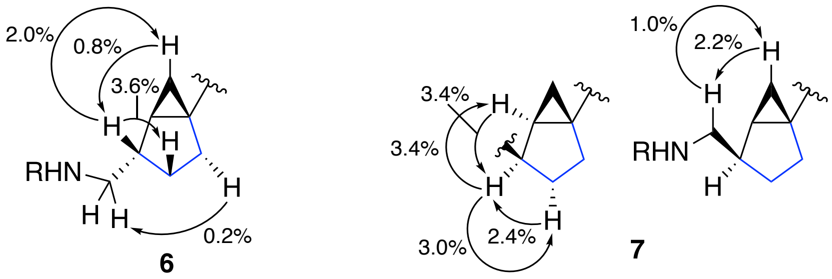

Figure S1. The observed NOEs in compounds **6** and **7** ($CDCl_3$, 500 MHz) for the determination of the configuration of the C3-position. The imidazole structures were deleted from this figures.

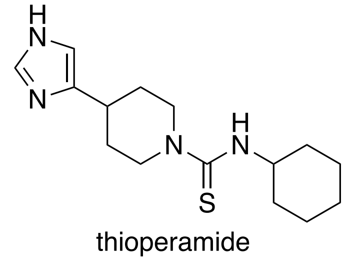

Figure S2. Chemical structure of thioperamide, which was used as an control in the binding assay.

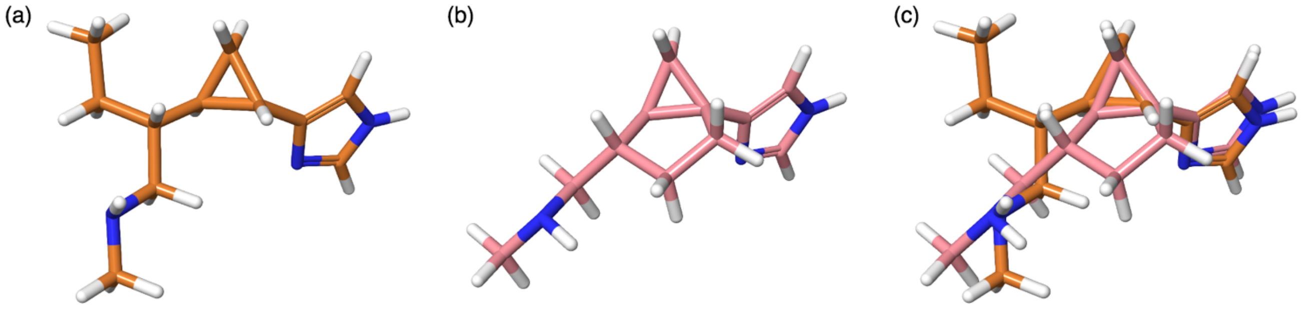

Figure S3. The most stable conformation of **4** (a) and **6** (b) by conformational search using MacroModel 10.9. (force field: MMFFs, solvent: H_2O); (c) Superimposition of the stable conformation of **4** and **6**. The chlorobenzyl moiety was replaced with a methyl group to simplify the structures.

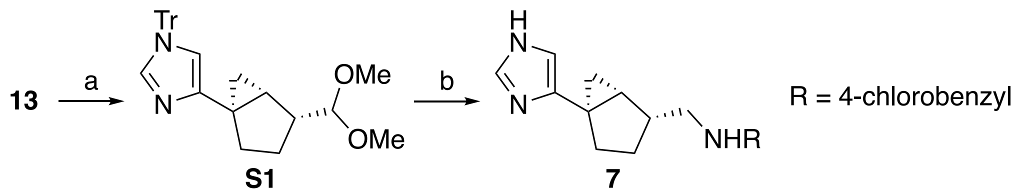

Scheme S1. Reagents and conditions: (a) (1) $TsCH_2NC$, NaOEt, EtOH, 0 °C; (2) sat. NH_3/EtOH, 125 °C, sealed tube; (3) TrCl, Et_3N, CH_2Cl_2, 33% in 3 steps; (b) (1) HCO_2H, hexane (2) 4-chlorobenzyl amine, $NaBH(OAc)_3$, CH_2Cl_2, MS4Å; (3) Boc_2O, Et_3N, DMAP, MeOH; (4) aq. HCl, EtOH, reflux, 16% in 4 steps.

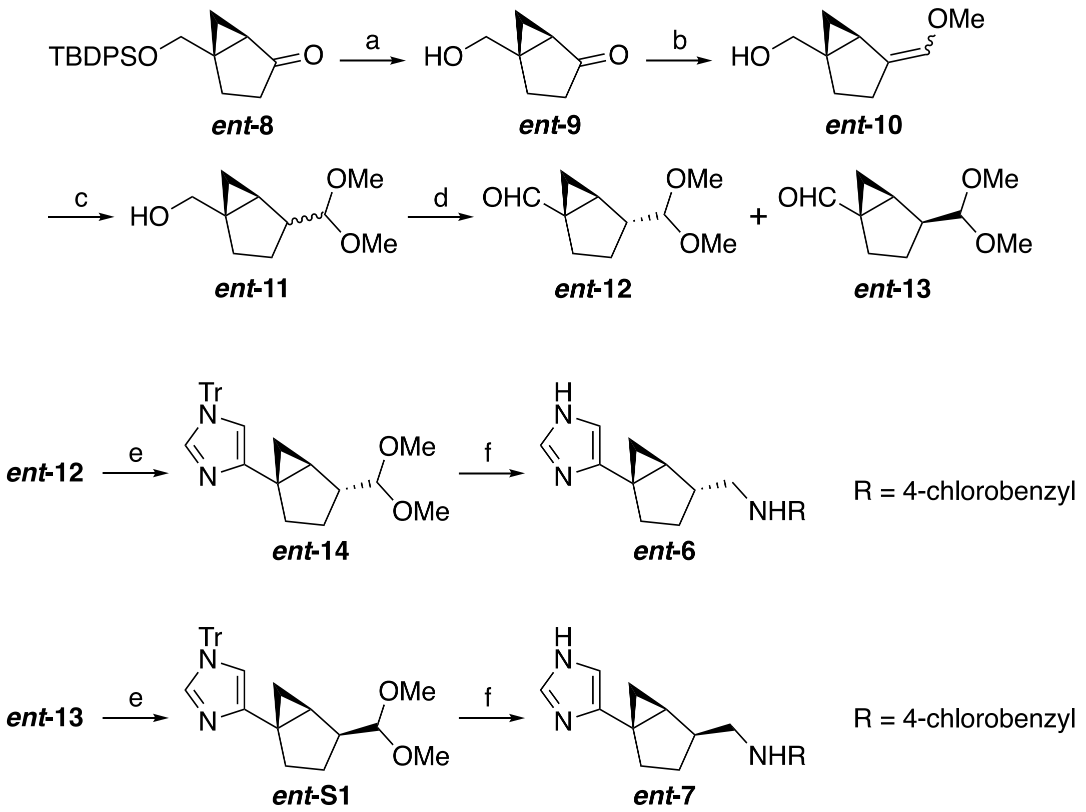

Scheme S2. Reagents and conditions: (a) $3HF{\bullet}Et_3N$, THF, 92%; (b) $MeOCH_2PPh_3Cl$, *t*BuOK, THF, 0 °C, 93%; (c) $TsOH{\bullet}H_2O$, MeOH, reflux, 63%; (d) $SO_3{\bullet}$pyridine, Et_3N, DMSO, 37% (***ent*-12**) and 43% (***ent*-13**); (e) (1) $TsCH_2NC$, NaOEt, EtOH, 0 °C; (2) sat. NH_3/EtOH, 125 °C, sealed tube; (3) TrCl, Et_3N, CH_2Cl_2, 59% (***ent*-14**) and 18% (***ent*-S1**) in 3 steps, respectively; (f) (1) HCO_2H, hexane (2) 4-chlorobenzyl amine, $NaBH(OAc)_3$, CH_2Cl_2, MS4Å; (3) Boc_2O, Et_3N, DMAP, MeOH; (4) aq. HCl, EtOH, reflux, 54% (***ent*-6**) and 47% (***ent*-7**) in 4 steps, respectively.

(1S,4R,5R)-4-Dimethoxymethyl-1-(1-triphenylmethyl-1H-imidazol-4-yl)bicyclo[3.1.0]hexane (**S1**).

S1 (58 mg, 0.12 mmol, 33% in 3 steps, pale yellow oil) was prepared from **13** (69 mg, 0.37 mmol) as described for the preparation of **14**. ^1H NMR (500 MHz, $CDCl_3$) δ 7.33–7.31 (10 H, m, aromatic), 7.14–7.12 (6 H, m, aromatic), 6.52 (1 H, s, imidazole-5), 4.16 (1 H, d, J = 8.0 Hz, -CHC<u>H</u>(OMe)$_2$), 3.39 (3 H, s, -OC<u>H$_3$</u>), 3.32 (3

H, s, -OC$\underline{H_3}$), 2.63 (1 H, m, H-4), 2.05 (1 H, m, H-3a), 1.95 (1 H, dd, J = 12.6, 8.0 Hz, H-2a), 1.70 (1 H, m, H-2b), 1.60 (1 H, m, H-5), 1.10 (1 H, m, H-3b), 0.98 (1 H, dd, J = 8.0, 4.6 Hz, H-6a), 0.85 (1 H, dd, J = 4.6, 4.6 Hz, H-6b); ^{13}C NMR (125 MHz, $CDCl_3$) δ 144.52, 142.47, 138.21, 129.78. 127.94 (Si-C), 127.92 (Si-C), 116.36, 107.69, 75.11, 53.18, 52.94, 42.52, 30.06, 27.54, 26.74, 23.62, 12.14; HRMS (ESI) calcd for $C_{31}H_{32}N_2NaO_2$ 487.2356, found 487.2340 [(M + Na)$^+$].

(1S,4R,5R)-4-[N-(4-Chlorobenzyl)aminomethyl]-1-(1H-imidazol-4-yl)bicyclo[3.1.0]hexane dihydrochloride (**7•2HCl**).

7•2HCl (6 mg, 15 μmol, 16% in 4 steps, hygroscopic white solid) was prepared from **S1** (24 mg, 52 μmol) as described for the preparation of **6•2HCl**. $[\alpha]_D^{29}$ = +43.1° (*c* 0.58, CH_3OH); ^{1}H NMR (500 MHz, CD_3OD) δ 8.78 (1 H, s, imidazole-2), 7.58 (2 H, d, J = 8.3 Hz, aromatic), 7.49 (2 H, d, J = 8.3 Hz, aromatic), 7.36 (1 H, s, imidazole-5), 4.28 (2 H, s, benzyl), 3.10 (2 H, d, J = 6.9 Hz, -CHC$\underline{H_2}$N-), 2.84 (1 H, m, H-4), 2.18–2.14 (2 H, m, H-2a and H-3a), 1.99–1.89 (2 H, m, H-2b and H-5), 1.20–1.12 (2 H, m, H-3b and H-6a), 1.03 (1 H, m, H-6b); ^{13}C NMR (125 MHz, CD_3OD) δ 138.26, 136.77, 134.71. 133.04, 131.17, 130.34, 116.37, 52.02, 51.32, 38.49, 31.84, 29.56, 26.47, 25.31, 12.43; HRMS (ESI) calcd for $C_{17}H_{21}N_3Cl$ 302.1419, found 302.1410 [(M + H)$^+$]. Anal. Calcd for $C_{17}H_{20}N_3Cl$•2.5HCl•0.1H_2O: C, 51.72; H, 5.85; N, 10.64. Found: C, 51.70; H, 5.85; N, 10.70.

(1R,5R)-1-Hydroxymethyl-bicyclo[3.1.0]hexan-4-one (***ent*-9**).

***ent*-9** (307 mg, 2.43 mmol, 92%, colorless liquid) was prepared from ***ent*-8** [1] (964 mg, 2.64 mmol) as described for the preparation of **9**. $[\alpha]_D^{210}$ = –4.2° (*c* 1.06, $CHCl_3$); HRMS (ESI) calcd for $C_{23}H_{28}NaO_2Si$ 387.1751, found 387.1748 [(M + Na)$^+$].

(1R,5R)-1-Hydroxymethyl-4-methoxymethylenebicyclo[3.1.0]hexane (***ent*-10**, *E*/*Z* mixture).

***ent*-10** (302 mg, 1.96 mmol, 93%, pale yellow oil) was prepared from ***ent*-9** (265 mg, 2.10 mmol) as described for the preparation of **10**. $[\alpha]_D^{21}$ = –46.6° (*c* 1.06, $CHCl_3$); HRMS (ESI) calcd for $C_9H_{14}NaO_2$ 177.0886, found 177.0887 [(M + Na)$^+$].

(1R,5S)-4-Dimethoxymethyl-1-hydroxymethylbicyclo[3.1.0]hexane (***ent*-11**, diastereomixture).

***ent*-11** (811 mg, 4.35 mmol, 63%, pale yellow liquid) was prepared from ***ent*-10** (1.06 g, 6.87 mmol) as described for the preparation of **11**. $[\alpha]_D^{21}$ = –53.4° (*c* 1.20, $CHCl_3$); HRMS (ESI) calcd for $C_{10}H_{18}NaO_3$ 209.1148, found 209.1149 [(M + Na)$^+$].

(1R,4R,5S)-4-Dimethoxymethyl-1-formylbicyclo[3.1.0]hexane (***ent*-12**, *anti*) and *(1R,4S,5S)-4-Dimethoxymethyl-1-formylbicyclo[3.1.0]hexane* (***ent*-13**, *syn*).

***ent*-12** (64 mg, 0.35 mmol, 37%, *anti,* less polar, colorless oil) and ***ent*-13** (75 mg, 0.41 mmol, 43%, *syn*, more polar, colorless oil) were prepared from ***ent*-11** (175 mg, 0.94 mmol) as described for the preparation of **12** and **13**. ***ent*-12**: $[\alpha]_D^{21}$ = +3.1° (*c* 1.04, $CHCl_3$); HRMS (ESI) calcd for $C_{10}H_{16}NaO_3$ 207.0992, found 207.0988 [(M

+ Na)$^+$]; ***ent*-13**: $[\alpha]_D^{21}$ = –72.1° (*c* 1.00, $CHCl_3$); HRMS (ESI) calcd for $C_{10}H_{16}NaO_3$ 207.0992, found 207.1001 [(M + Na)$^+$].

(1R,4R,5S)-4-Dimethoxymethyl-1-(1-triphenylmethyl-1H-imidazol-4-yl)bicyclo[3.1.0]hexane (***ent*-14**).
***ent*-14** (87 mg, 0.19 mmol, 59% in 3 steps, pale yellow oil) was prepared from ***ent*-12** (59 mg, 0.32 mmol) as described for the preparation of **14**. HRMS (ESI) calcd for $C_{31}H_{32}N_2NaO_2$ 487.2356, found 487.2372 [(M + Na)$^+$].

(1R,4R,5S)-4-[N-(4-Chlorobenzyl)aminomethyl]-1-(1H-imidazol-4-yl)bicyclo[3.1.0]hexane dihydrochloride (***ent*-6•2HCl**).
***ent*-6•2HCl** (14 mg, 37 μmol, 54% in 4 steps, hygroscopic white solid) was prepared from ***ent*-14** (32 mg, 68 μmol) as described for the preparation of **6•2HCl**. $[\alpha]_D^{29}$ = –16.8° (*c* 0.74, CH_3OH); HRMS (ESI) calcd for $C_{17}H_{21}N_3Cl$ 302.1419, found 302.1438 [(M + H)$^+$]. Anal. Calcd for $C_{17}H_{20}N_3Cl$•2.2HCl: C, 53.45; H, 5.86; N, 11.00. Found: C, 53.33; H, 5.85; N, 10.91.

(1R,4S,5S)-4-Dimethoxymethyl-1-(1-triphenylmethyl-1H-imidazol-4-yl)bicyclo[3.1.0]hexane (***ent*-S1**).
***ent*-S1** (33 mg, 0.071 mmol, 18% in 3 steps, pale yellow oil) was prepared from ***ent*-13** (74 mg, 0.40 mmol) as described for the preparation of **14**. HRMS (ESI) calcd for $C_{31}H_{32}N_2NaO_2$ 487.2356, found 487.2364 [(M + Na)$^+$].

(1R,4S,5S)-4-[N-(4-Chlorobenzyl)aminomethyl]-1-(1H-imidazol-4-yl)bicyclo[3.1.0]hexane dihydrochloride (***ent*-7•2HCl**).
***ent*-7•2HCl** (12 mg, 32 μmol, 47% in 4 steps, hygroscopic white solid) was prepared from ***ent*-S1** (31 mg, 67 μmol) as described for the preparation of **6•2HCl**. $[\alpha]_D^{29}$ = –49.0° (*c* 0.30, CH_3OH); HRMS (ESI) calcd for $C_{17}H_{21}N_3Cl$ 302.1419, found 302.1400 [(M + H)$^+$]. Anal. Calcd for $C_{17}H_{20}N_3Cl$•2HCl•0.3H_2O: C, 53.71; H, 5.99; N, 11.05. Found: C, 53.70; H, 5.91; N, 11.05.

Reference

1. Kobayashi, T.; Suemasa, A.; Igawa, A.; Ide, S.; Fukuda, H.; Abe, H.; Arisawa, M.; Minami, M.; Shuto, S. Conformationally restricted GABA with bicyclo[3.1.0]hexane backbone as the first highly selective BGT-1 inhibitor. *ACS. Med. Chem. Lett.* **2014**, *5*, 889–893.

6•2HCl

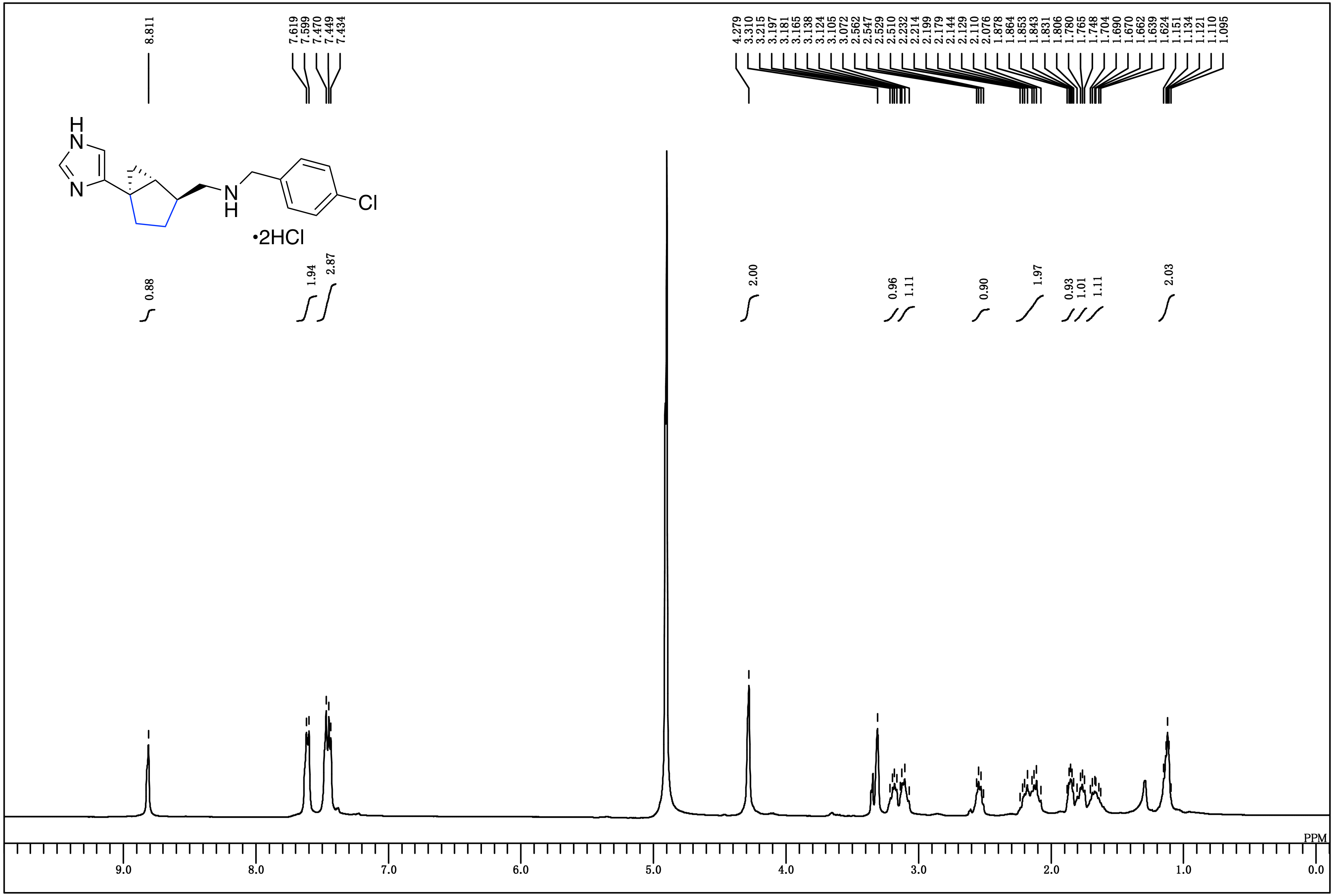
8.811
7.619
7.599
7.470
7.449
7.434
4.279
3.310
3.215
3.197
3.181
3.165
3.138
3.124
3.105
3.072
2.562
2.547
2.529
2.510
2.232
2.214
2.199
2.179
2.144
2.129
2.110
2.076
1.878
1.864
1.853
1.843
1.831
1.806
1.780
1.765
1.748
1.704
1.690
1.670
1.662
1.639
1.624
1.151
1.134
1.121
1.110
1.095
Cl
•2HCl
0.88
1.94
2.87
2.00
0.96
1.11
0.90
1.97
0.93
1.01
1.11
2.03
PPM
9.0
8.0
7.0
6.0
5.0
4.0
3.0
2.0
1.0
0.0

ent-6•2HCl

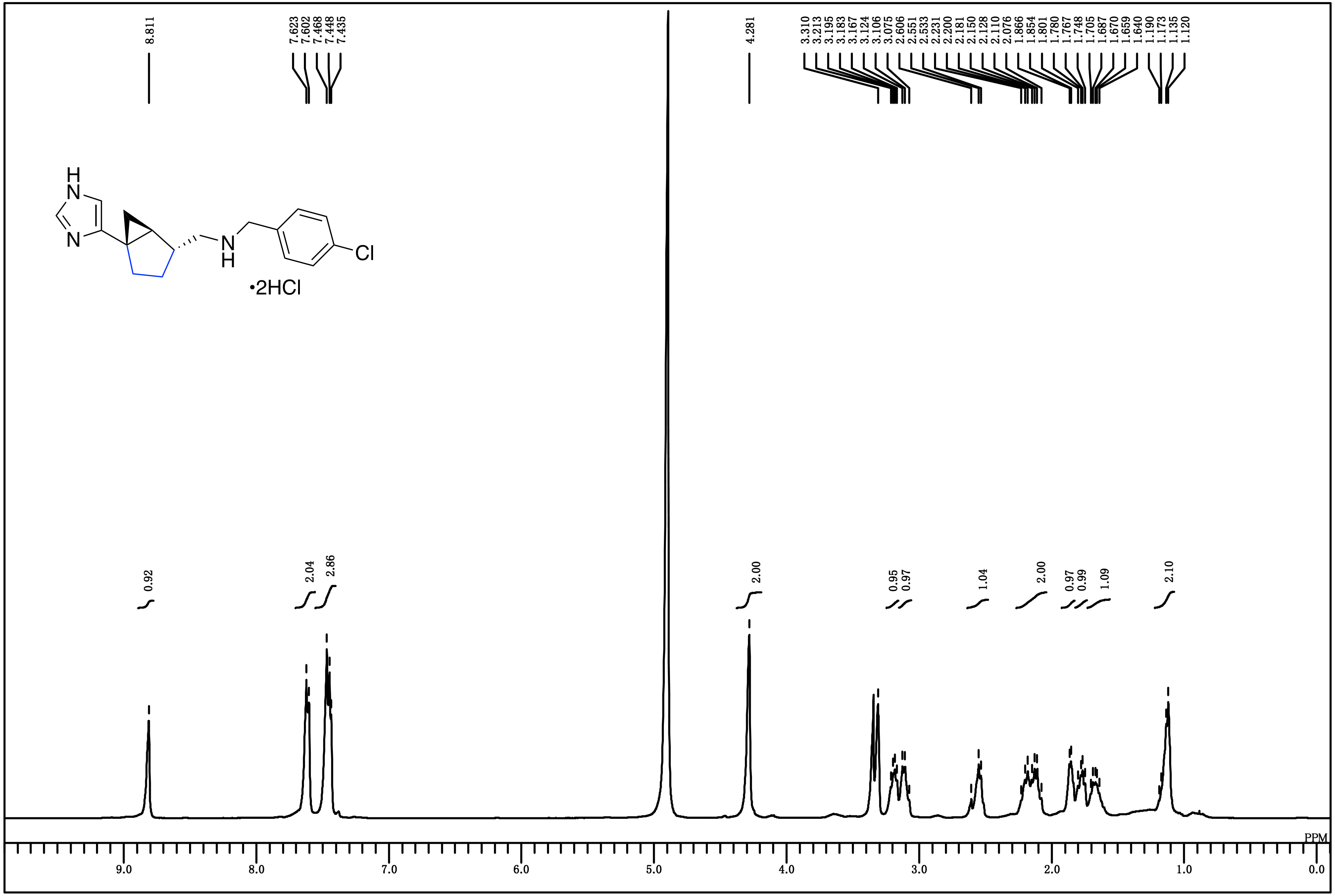
8.811
7.623
7.602
7.468
7.448
7.435
4.281
3.310
3.213
3.195
3.183
3.167
3.124
3.106
3.075
2.606
2.551
2.533
2.231
2.200
2.181
2.150
2.128
2.110
2.076
1.866
1.854
1.801
1.780
1.767
1.748
1.705
1.687
1.670
1.659
1.640
1.190
1.173
1.135
1.120
H
N
N
N
H
Cl
•2HCl
0.92
2.04
2.86
2.00
0.95
0.97
1.04
2.00
0.97
0.99
1.09
2.10
PPM
9.0
8.0
7.0
6.0
5.0
4.0
3.0
2.0
1.0
0.0

7•2HCl

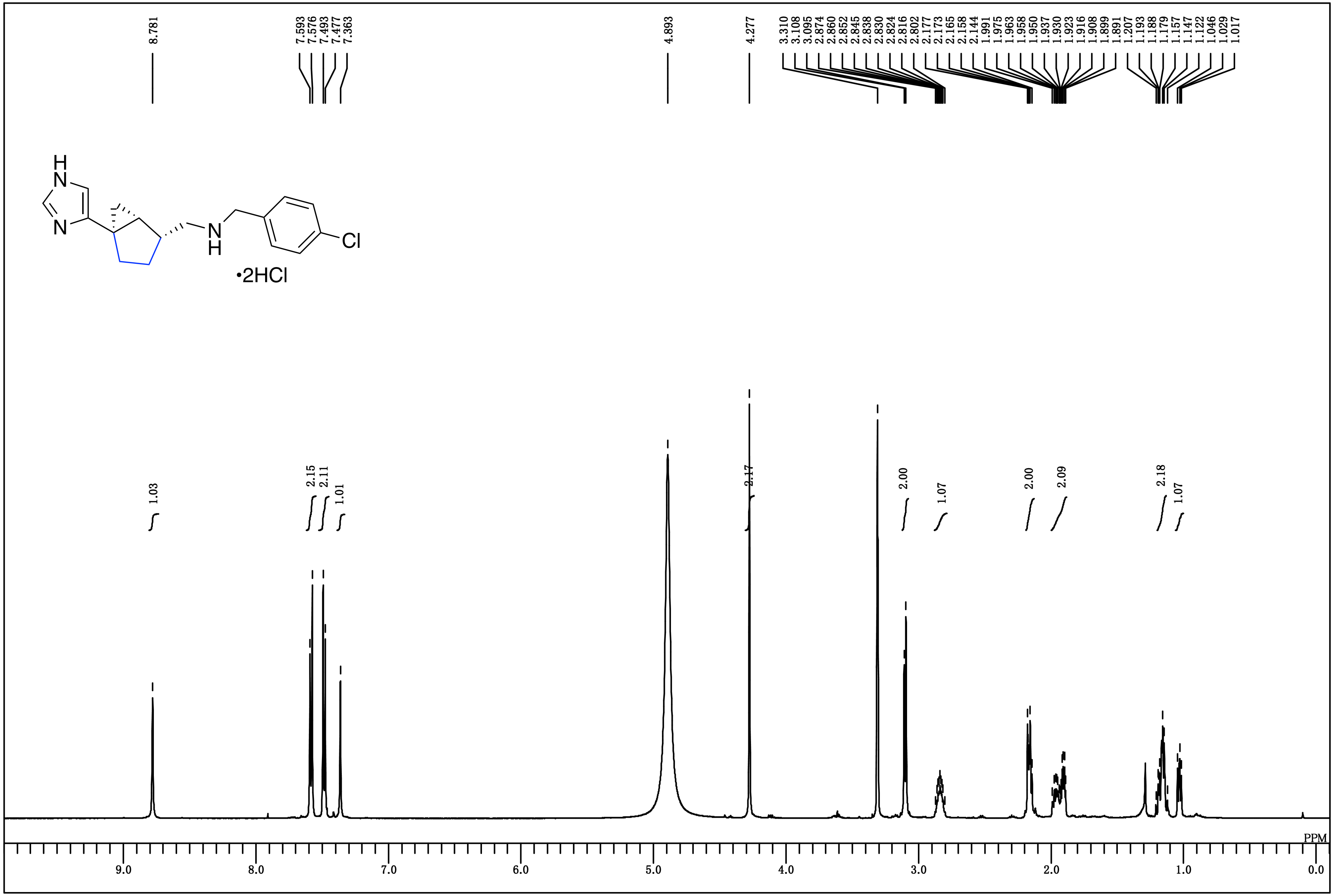
8.781
7.593
7.576
7.493
7.477
7.363
4.893
4.277
3.310
3.108
3.095
2.874
2.860
2.852
2.845
2.838
2.830
2.824
2.816
2.802
2.177
2.173
2.165
2.158
2.144
1.991
1.975
1.963
1.958
1.950
1.937
1.930
1.923
1.916
1.908
1.899
1.891
1.207
1.193
1.188
1.179
1.157
1.147
1.122
1.046
1.029
1.017
1.03
2.15
2.11
1.01
2.17
2.00
1.07
2.00
2.09
2.18
1.07
H
N
N
N
H
Cl
•2HCl
PPM
9.0
8.0
7.0
6.0
5.0
4.0
3.0
2.0
1.0
0.0

ent-7•2HCl

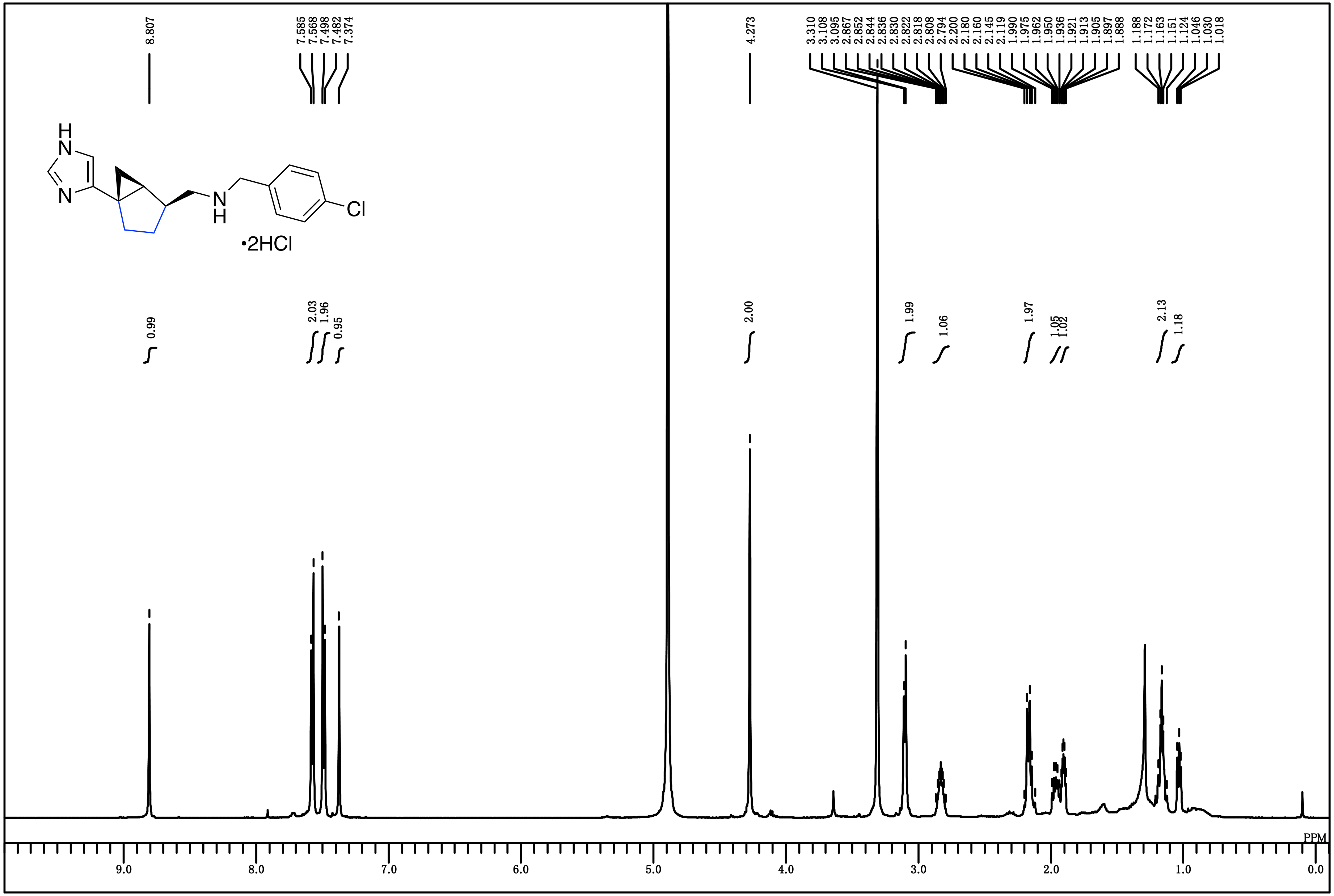
8.807
7.585
7.568
7.498
7.482
7.374
4.273
3.310
3.108
3.095
2.867
2.852
2.844
2.836
2.830
2.822
2.818
2.808
2.794
2.200
2.180
2.160
2.145
2.119
1.990
1.975
1.962
1.950
1.936
1.921
1.913
1.905
1.897
1.888
1.188
1.172
1.163
1.151
1.124
1.046
1.030
1.018
•2HCl
Cl
0.99
2.03
1.96
0.95
2.00
1.99
1.06
1.97
1.05
1.02
2.13
1.18
PPM
9.0
8.0
7.0
6.0
5.0
4.0
3.0
2.0
1.0
0.0
